# A Green and Efficient Method for the Preconcentration and Determination of Gallic Acid, Bergenin, Quercitrin, and Embelin from* Ardisia japonica* Using Nononic Surfactant Genapol X-080 as the Extraction Solvent

**DOI:** 10.1155/2018/1707853

**Published:** 2018-02-01

**Authors:** Ying Chen, Kunze Du, Jin Li, Yun Bai, Mingrui An, Zhijing Tan, Yan-xu Chang

**Affiliations:** ^1^Tianjin State Key Laboratory of Modern Chinese Medicine, Tianjin University of Traditional Chinese Medicine, Tianjin 300193, China; ^2^Key Laboratory of Formula of Traditional Chinese Medicine, Tianjin University of Traditional Chinese Medicine, Ministry of Education, Tianjin 300193, China; ^3^Department of Surgery, University of Michigan, Ann Arbor, MI 48109, USA

## Abstract

A simple cloud point preconcentration method was developed and validated for the determination of gallic acid, bergenin, quercitrin, and embelin in* Ardisia japonica* by high-performance liquid chromatography (HPLC) using ultrasonic assisted micellar extraction. Nonionic surfactant Genapol X-080 was selected as the extraction solvent. The effects of various experimental conditions such as the type and concentration of surfactant and salt, temperature, and solution pH on the extraction of these components were studied to optimize the conditions of* Ardisia japonica*. The solution was incubated in a thermostatic water bath at 60°C for 10 min, and 35% NaH_2_PO_4_ (w/v) was added to the solution to promote the phase separation and increase the preconcentration factor. The intraday and interday precision (RSD) were both below 5.0% and the limits of detection (LOD) for the analytes were between 10 and 20 ng·mL^−1^. The proposed method provides a simple, efficient, and organic solvent-free method to analyze gallic acid, bergenin, quercitrin, and embelin for the quality control of* Ardisia japonica*.

## 1. Introduction

**Figure 1:**
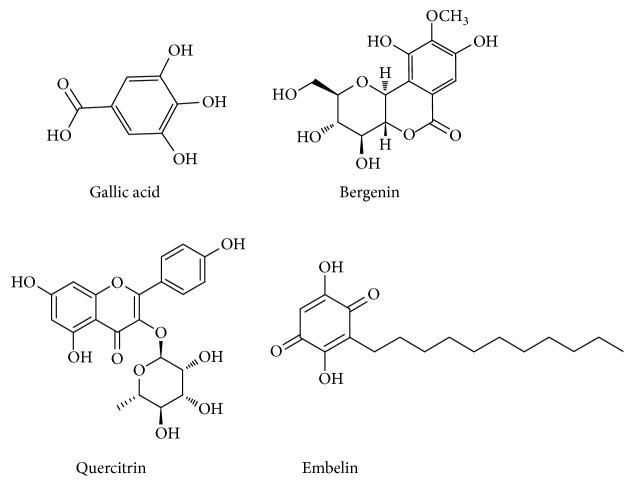
Chemical structures of gallic acid, bergenin, quercitrin, and embelin.

*Ardisia japonica*, one of common traditional Chinese medicines, belongs to the family of Ardisia [1].* Ardisia japonica* has many medicinal and ornamental values and has drawn a global attention [2, 3]. Recent studies have reported a myriad of chemical compounds, antibacterial activity, and pharmacological properties. Many compounds including gallic acid [4], bergenin [5], quercitrin, and embelin have been elucidated (Figure 1).* Ardisia japonica* has been used to cure pancreatic, pneumonia, bronchitis conjunctivitis, and trauma and other types of cancer [1]. It has also been proven to possess anticancer activity [6] and anti-HIV activity [7]. Therefore, it has become imperative to develop a method of separation and purification of the compounds presenting in* Ardisia japonica.*

Many analytical methods for the extraction of bioactive compounds are employed in sample preparation [8, 9], but it is very challenging to determine trace bioactive compounds at low concentrations in the matrixes. It is therefore necessary to separate and concentrate the targeted analytes from the matrix sample to improve the detection sensitivity [10]. Many papers have reported enrichment methods including free air carbon dioxide enrichment (FACE) [11], cloud point extraction (CPE) [12], ionic liquid/ionic liquid liquid-liquid microextraction [13], solid-phase extraction (SPE) [14], and aqueous two-phase system (ATPS) [15]. The micelle-mediated extraction and cloud point preconcentration method provide a suitable alternative to the common extraction methods [16]. The surfactant-rich phase is a small volume of with a high concentration of surfactant and the aqueous phase is a large volume with a low concentration of surfactant. The aqueous phase is discarded while the supernatant (surfactant-rich phase) was leaved and dissolved by methanol. Compared with common extraction methods, cloud point extraction (CPE) bears many advantages such as less cost, smaller aliquot of organic solutions, less toxicity, and higher precision. Thus, it is regarded as a rapid eco-friendly technique with the high precision due to the small volume of surfactant-rich phase and the reduction in the use of toxic organic solvents. At present, CPE has been used in different areas of extraction and preconcentration, including blood [17, 18], urine [19], water [18], food [20], metal ions [21], rare earth elements [22], and Chinese herbs [23]. Considering the complex matrixes of Chinese herbs, a micelle-mediated extraction and cloud point preconcentration method is fast becoming important for separation and preconcentration of analytes in Chinese herbs.

**Figure 2:**
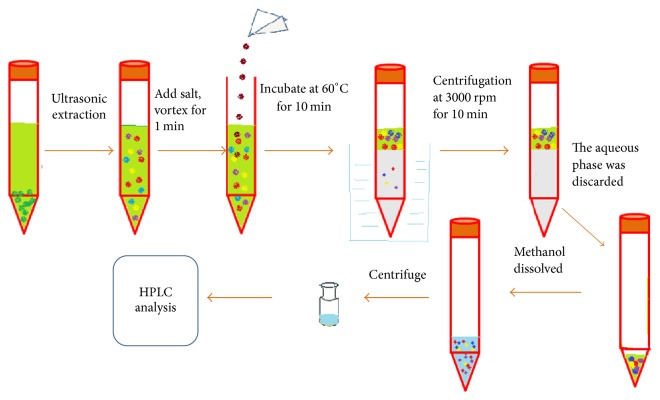
Schematic diagram for micellar extraction and cloud point preconcentration.

The aim of this study was to develop CPE method based on surfactants for extraction and preconcentration of gallic acid, bergenin, quercitrin, and embelin from* Ardisia japonica* samples (Figure 2). Through a series of optimum conditions, including the effects of ionic strength, pH of extraction solvent, bath temperature, and time which were all studied, the best conditions for* Ardisia japonica* samples were obtained. It was successfully employed to the quantification of gallic acid, bergenin, quercitrin, and embelin from* Ardisia japonica* samples.

## 2. Experimental

### 2.1. Plant Materials

The dried* Ardisia japonica* was obtained from a local pharmacy (China). The dried* Ardisia japonica* was pulverized using a grinder and sieved with a 100 mesh sieve to produce sample. The sample solutions were stored under controlled moisture and temperature.

### 2.2. Chemicals and Reagents

The standard of gallic acid, bergenin, quercitrin, and embelin (purity > 98%) were obtained from Chinese Academy of Sciences (Chengdu, China). Nonionic surfactant Genapol X-080 was purchased from Sigma (USA). Various concentrations (v/v) of Genapol X-080 solutions were prepared by dissolving appropriate amounts of Genapol X-080 in deionized water. Sodium dihydrogen phosphate, hydrochloric acid, and sodium hydroxide were of analytical grade and obtained from Beijing Chemical Factory (Beijing, China); acetonitrile was HPLC-grade and obtained from Fisher (Leicestershire, UK). All other reagents used in this work were of analytical grade. Deionized water (Milli-Q) was used throughout this study.

### 2.3. Preparation of Standard Solutions

The stock standard solutions of gallic acid, bergenin, quercitrin, and embelin (1.0 mg mL^−1^) were made by dissolving appropriate amounts of these compounds in methanol. Then, the working standard solution was obtained by mixing certain concentration of the four stock solutions together and diluted with 4% Genapol X-080.

### 2.4. Instrumentation

All analysis was performed on Waters HPLC system (2695 series). The HPLC system consisted of a quaternary pump, a photodiode array detector and a column thermostat. The analytical column was an Ultimate XB-C18 (250 mm × 4.6 mm i.d, 5 *μ*m) connected to an Agilent Zorbax Extend-C18 guard column (12.5 mm × 2.1 mm i.d., 5 *μ*m). The column temperature was 35°C. Chromatographic data processing was aided by Empower software in HPLC systems.

Samples were pulverized and sieved (Zhejiang, China). A vortex mixer (WH-861, Taicang, China), a pH meter (Crison pH 2000, Shanghai, China), and a thermostatic bath (HH-2, Guangzhou, China) were used to performance cloud point extraction. High-speed centrifugation (Sigma) was applied to increase the phase separation process.

### 2.5. Experimental Procedures

#### 2.5.1. Micelle-Mediated Extraction Procedure

The dried* Ardisia japonica* powder (0.1000 g) was accurately weighed and put it into a 50 mL centrifuge tube. 20 mL Genapol X-080 solution (4%) (v/v) was then added. The powders and Genapol X-080 solution (4%) (v/v) were thoroughly mixed and then were extracted (400 W) for 40 min at room temperature (22°C) in the ultrasonic cleaning bath. The extract solution was centrifuged at 5000 rpm for 10 min and the supernatant was filtered through a 0.45 *μ*m membrane.

#### 2.5.2. Cloud Point Preconcentration

10 mL supernatant of extract was accurately measured and placed in a 15 mL centrifuge tube. 35% (w/v) sodium dihydrogen phosphate was diluted by the supernatant and the solution pH was adjusted to 5 by 10% of HCL and 0.1 M NaOH solutions in a test tube. The sodium dihydrogen phosphate was added to the sample solution and dissolved thoroughly with a Vortex Genie Mixture for 1 min and then incubated in the thermostatic bath at 60°C for 10 min. The phase separation was accelerated by centrifugation at 3000 rpm for 10 min. The aqueous phase was then discarded, leaving the surfactant-rich phase of volume 600 *μ*L. The surfactant-rich phase was dissolved in methanol and topped up to 2 mL using a glass volumetric flask. After ultrasonic processing for 3 min and centrifuging at 14000 r min^−1^ for 10 min, a 0.45 *μ*m nylon membrane was applied to filter the samples. 10 *μ*L of the solution was injected into the HPLC system for analysis.

### 2.6. HPLC Analysis

0.1% formic acid (A) and acetonitrile (B) was selected as mobile phase. The linear gradient was 5–9% B over 0–4 min, 9–14% B over 4–7 min, 14–30% B over 7–20 min, 30–60% B over 20–25 min, 60–86% B over 25–30 min, 86–100% B over 30–40 min, and 100% B over 40–45 min and then returned to 5% B at 46 min immediately. The flow rate was 1.0 mL min^−1^. The detector was at 275 nm and the column temperature was maintained at 35°C. In order to avoid the influence of salt and Genapol X-080 to the separation of analytes and to protect the column, acetonitrile and water were used to sufficiently elute.

## 3. Results and Discussion

### 3.1. Optimization of Micellar Extraction

In order to optimize the CPE of gallic acid, bergenin, quercitrin, and embelin from the herbal sample, a series of experiments including the type of surfactant, surfactant concentration, ultrasonic extracting time, ultrasonic extraction power, solid-liquid rate, ionic strength, pH of sample solution, bath temperature, and time under different conditions were studied. The peak area of the analyte was used to evaluate the effect of these factors.

#### 3.1.1. Selection of the Surfactant in Micellar Extraction

**Figure 3:**
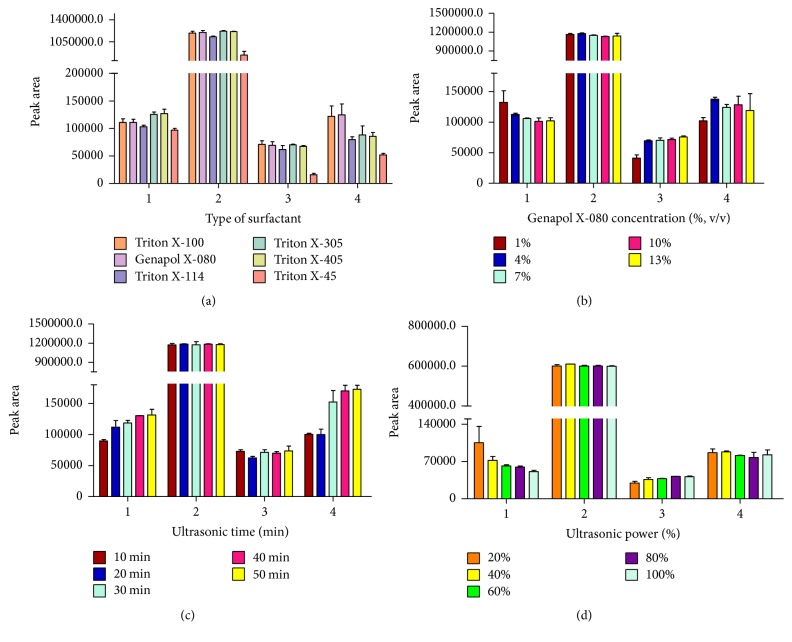
Effect of some parameters of ultrasonic assisted micellar extraction on the extraction effect of 4 components: (a) type of surfactant; (b) Genapol X-080 concentration (%, v/v); (c) ultrasonic time (min); (d) ultrasonic power (%). 1, gallic acid; 2, bergenin; 3, quercitrin; 4, embelin.

The type of surfactant plays an important role on micellar extraction. Seven surfactants (Triton X-100, Triton X-114, Triton X-305, Triton X-405, Triton X-45, and Genapol X-080) were optimized as extraction solvents (Figure 3(a)). It was found that highest peak areas of analytes were obtained when Genapol X-080 and Triton X-100 were selected as extraction solvents. Compared with Triton X series surfactant, the Genapol X-080 does not absorb at 275 nm by HPLC-UV method. In addition, Genapol X-080 is the inexpensive and eco-friendly surfactant [24]. Therefore, Genapol X-080 was chosen as the CPE surfactant for next step in this research.

#### 3.1.2. Genapol X-080 Concentration in Micellar Extraction

Genapol X-080 concentration was studied in the range of 1–13% (v/v). Other conditions were kept constant, including 20 mL Genapol X-080 as extraction solvent and 40 min as ultrasound time. The critical micellar concentration (CMC) of Genapol X-080 is 0.05 mM (2.9%, v/v) [25]. When the concentration of surfactant is at 1% (below CMC), it is difficult to form two phases. When the Genapol X-080 concentration increases to 4%, the peak areas of analytes were highest. When the Genapol X-080 concentration continues to rise, the solution becomes too sticky to be handled so that the peak area of analytes would not increase. The character of the aqueous nonionic Genapol X-080 solution could increase the solubility of analytes via the surfactant micelles. According to above-mentioned results (Figure 3(b)), 4% Genapol X-080 was selected for obtaining highest peak areas.

#### 3.1.3. Ultrasonic Extraction Time Effect in Micellar Extraction

The influence of ultrasonic time on extraction effect of gallic acid, bergenin, quercitrin, and embelin were studied by changing the ultrasonic time from 10 to 50 min (Figure 3(c)). The results indicated that the amount of extracted gallic acid, bergenin, quercitrin, and embelin increased with the increase of extraction time and the extraction yield of gallic acid, quercitrin, and embelin reached the highest value at 50 min. There was no significant difference between the peak areas of the four analytes extracted by ultrasonic extraction for 50 min and those extracted by ultrasonic extraction for 40 min (*P* > 0.05). Hence, 40 min was chosen for the ultrasonic extraction of gallic acid, bergenin, quercitrin, and embelin.

#### 3.1.4. Ultrasonic Power Effect in Micellar Extraction

The effect of ultrasonic power on extraction ability of gallic acid, bergenin, quercitrin, and embelin were studied by varying the ultrasonic power from 20% to 100% at the optimal condition (Figure 3(d)). For bergenin and embelin, the peak area reached the highest under the ultrasonic power of 40%. For gallic acid and quercitrin, the peak area reached the highest under the ultrasonic power of 20% and 80%, respectively. The result of this method is consistent with the reported research that ultrasound can improve diffusion and then increases the extract yield of analytes [26]. There is no difference between the peak area of gallic acid under the ultrasonic power 20% and that under the ultrasonic power 40% (*P* > 0.05). Consequently, 40% of the ultrasonic power was chosen for the extraction of the sample.

#### 3.1.5. Liquid/Solid Ratio Effect in Micellar Extraction

**Figure 4:**
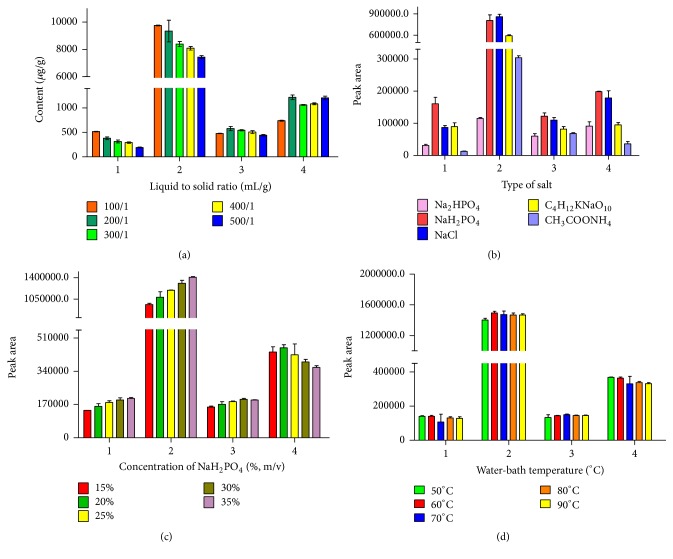
Effect of some parameters of cloud point extraction on the extraction effect of 4 components: (a) liquid to solid ratio (mL/g); (b) type of salt; (c) concentration of NaH2PO4 (%, m/v); (d) water-bath temperature (°C). 1, gallic acid; 2, bergenin; 3, quercitrin; 4, embelin.

Liquid/solid ratio is the ratio between the volume of solvent and the amount of crude material. The effects of liquid/solid ratio on extraction ability of gallic acid, bergenin, quercitrin, and embelin were studied by varying the liquid/solid ratio from 100 : 1 to 500 : 1 (mL g^−1^) at the optimal condition above (Figure 4(a)). The extraction yield of quercitrin and embelin was the highest under the liquid/solid ratio at 200 : 1 (20 mL/0.1 g). The extraction yield of gallic acid and bergenin was the highest under the liquid/solid ratio at 100 : 1 (10 mL/0.1 g). There was no significant difference between the peak areas the extraction yield of the bergenin at the liquid/solid ratio of 100 : 1 and those extracted by at the liquid/solid ratio of 200 : 1 (*P* > 0.05). Considering the extraction efficiency of embelin and bergenin, the liquid/solid ratio of 200 : 1 (mL g^−1^) was used to perform the following experiments.

The optimal condition was obtained from micellar extraction by serious of experiment above: 0.1 g of the powders and 20 mL Genapol X-080 solution (4%) (v/v) were thoroughly mixed and then put through ultrasonic extraction (400 W) for 40 min.

### 3.2. Optimization of Cloud Point Preconcentration

#### 3.2.1. The Effect of the Type of Salt

The type of salt is a key factor in CPE. The addition of salt could promote the separation of surfactant from water [25]. It is one of the factors influencing the cloud point extraction efficiency of gallic acid, bergenin, quercitrin, and embelin. Supernatant was obtained from the optimal condition of micellar extraction. Five additions (NaH_2_PO_4_, Na_2_HPO_4_, NaCl, CH_3_COONH_4_, and C_4_H_12_KNaO_10_) were chosen to optimize the conditions. As shown in Figure 4(b), the highest extract efficiency of gallic acid, quercitrin, and embelin was obtained when NaH_2_PO_4_ was selected as addition salt. In addition, it was found that there is no significant difference (*P* > 0.05) for the extract of bergenin between NaH_2_PO_4_ and NaCL (Figure 4(b)). Thus, NaH_2_PO_4_ was employed in the following experiments.

#### 3.2.2. The Effect of Concentration of NaH_2_PO_4_

The concentration of sodium dihydrogen phosphate is also a key factor in CPE [27]. The addition of salt to the sample solution can influence the extraction resulting from a changing in density of the aqueous phase [28]. The influence of the ionic strength on the extraction efficiency was carried out by changing the concentration of sodium dihydrogen phosphate between 15% and 35% (w/v). The results showed that the addition of sodium dihydrogen phosphate promoted the separation between the surfactant-rich phase and the aqueous phase. With the salt concentration increased, the micelle size and the aggregation number were also increasing and analytes might become more soluble in the surfactant-rich phase, while the critical micellar concentration stayed constant [29]. Therefore, the addition of salt not only promoted the transform hydrophilic compounds into the surfactant-rich phase, but also reduced the volume of the surfactant-rich phase, most likely via some type of dehydration mechanism [30–32]. In this method, 35% sodium dihydrogen phosphate was applied. The results in Figure 4(c) indicated that the CPE at a salt concentration of 35% (w/v) obtained the optimum extraction recovery of gallic acid and bergenin. Higher peak area of quercitrin was obtained at the salt concentration of 30% (w/v). Although the highest peak area of embelin was obtained at the salt concentration of 20% (w/v), 35% sodium dihydrogen phosphate was selected as addition to rich the four analytes according to other three compounds.

#### 3.2.3. The Effect of Incubation Temperature and Time

**Figure 5:**
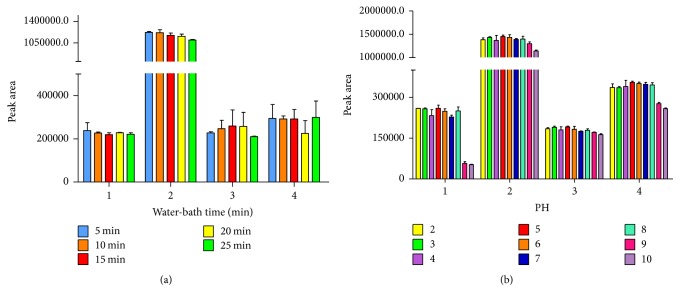
Effect of water-bath time (a) and pH of cloud point extraction (b) on the extraction effect of 4 components. 1, gallic acid; 2, bergenin; 3, quercitrin; 4, embelin.

Incubation temperature and time are two important factors of the cloud point extraction. The aqueous solution of surfactant divides into two phases when at certain temperature, called cloud point temperature (CPT) of the surfactant [33]. The optimal incubation temperature for the extraction was obtained by 15–20°C, which was higher than the cloud point of the surfactant (42°C in pure water [25]). The influence of temperature on the extraction effect was studied in the range of 50–90°C (Figure 4(d)). The results showed that the highest peak areas of gallic acid, bergenin, and embelin were obtained at 60°C and the highest peak area of quercitrin was obtained at 50°C. It was found that there no significant different between peak area of quercitrin at 60°C and that at 50°C. Therefore, 60°C was employed as optimized incubation temperature. The effect of different incubation time on the extraction efficiency was studied by varying the incubation time from 5 to 25 min (Figure 5(a)). The results indicated that higher peak areas of gallic acid and embelin were obtained at the incubation time of 5 min while higher peak areas of bergenin and quercitrin were obtained at the incubation time of 10 min. A slight decrease of peak area of three analytes (gallic acid, bergenin, and quercitrin) was also found when the extraction time incubation was longer than 20 min. There was no significance between peak areas of gallic acid and embelin at 60°C for 10 min and those at 60°C for 5 min (*P* < 0.05). Therefore, the CPE was performed at 60°C for 10 min.

#### 3.2.4. The Effect of pH

It was well-known that partition of some ionizable organic analytes in two immiscible phases depends on solution pH. The effect of pH on the analytes extraction efficiency was studied over the range of 2–10 and adjusted by 10% HCL and 0.01 M NaOH solution (Figure 5(b)). The results showed that the solution pH in acidic environment had no significant effect on the extraction of analytes while the pH of solution has the significant effect on the extraction of gallic acid and embelin in alkaline environment. This phenomenon might be due to the formation of ion-pairs of the analytes. It was found that the maximum extraction efficiency of four analytes was achieved at pH 5. Therefore, the CPE was performed at pH 5.

### 3.3. HPLC Profiles of the Extracted Four Compounds

**Figure 6:**
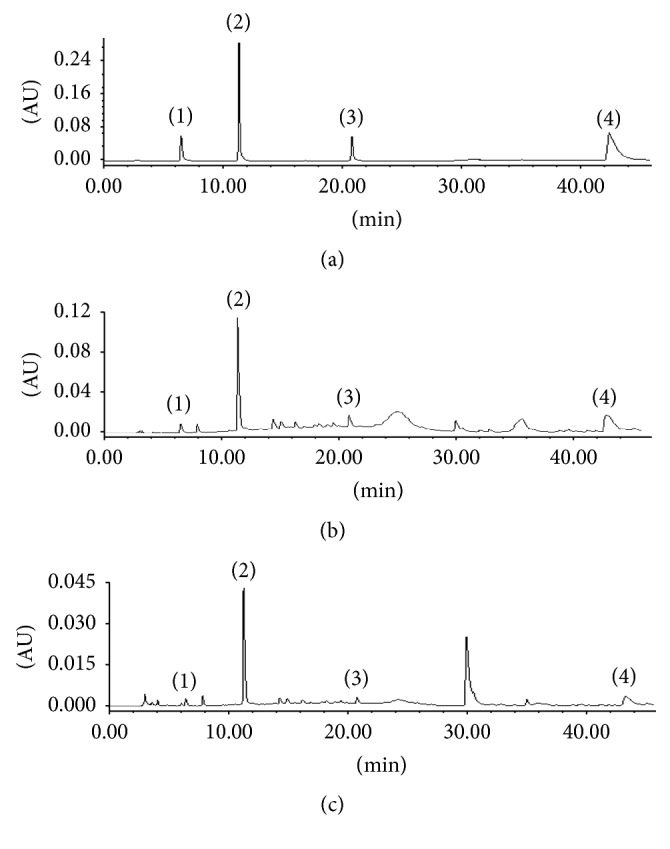
The typical HPLC chromatograms of (a) the mixed standard solutions: (1) gallic acid, 50 *μ*g mL^−1^; (2) bergenin, 500 *μ*g mL^−1^; (3) quercitrin, 50 *μ*g mL^−1^; (4) embelin, 250 *μ*g mL^−1^; (b) sample extracted by ultrasonic assisted micellar extraction and then preconcentrated by cloud point extraction: (1) gallic acid, 5.58 *μ*g mL^−1^; (2) bergenin, 216.65 *μ*g mL^−1^; (3) quercitrin, 12.72 *μ*g mL^−1^; (4) embelin, 48.45 *μ*g mL^−1^; (c) sample extracted by ultrasonic assisted micellar extraction. (1) gallic acid, 1.09 *μ*g mL^−1^; (2) bergenin, 39.93 *μ*g mL^−1^; (3) quercitrin, 2.59 *μ*g mL^−1^; (4) embelin, 10.46 *μ*g mL^−1^.

Based on the experiments result above, the optimum PCE conditions were as follows: 4% (v/v) Genapol X-080, 35% (w/v) NaH_2_PO_4_ in 10 mL solution (pH 5), and 10 min incubation at 60°C. Under the optimized conditions, enrichment factor of all the four compounds was greater. The results showed that the extraction efficiency of the analytes increased significantly.  Figure 6 showed the chromatogram of four compounds extracted from* Ardisia japonica* samples after mixed cloud point extraction. It could be observed that the chromatographic condition allowed a good separation of analytes.

### 3.4. Analytical Method Validation

At least six described concentrations of the standard solution extracted by ultrasonic assisted micellar extraction and then preconcentrated by cloud point extraction method were analyzed. Then the calibration curves were constructed by plotting the peak area versus the concentrations of compounds. Correlation coefficients of the calibration curves were found to be higher than 0.99. The regression data obtained showed good linear relationship. Limits of detection (LOD) for the four compounds were less than 16.0 ng mL^−1^ (*S*/*N* = 3), and limits of quantification (LOQ) were less than 40.0 ng mL^−1^ (*S*/*N* = 10), which showed a high sensitivity under these experimental conditions (Table 1).

The intraday precision and interday precision were studied by analyzing three different concentrations of standard solutions. The intraday variance was determined by analyzing the spiked samples six times at one day. The interday variance was studied for three continuous days. Variations were indicated by the RSD. The RSDs were all less than 5% for intraday and interday. The accuracies were determined by comparing the mean calculated concentration with the spiked target concentration of the three different concentrations samples. The intraday and interday accuracies for the four analytes were found to be within 95.9% and 100% (Table 2). The recovery of the method was determined using the standard addition method. The standard analytes were added to approximately 0.025 g of the* Ardisia japonica* extract sample and then extracted and analyzed using the method described above. The total content of each analyte was calculated by the corresponding calibration curve. The formula was used to calculate the recoveries: recovery (%) = (amount found − original amount)/amount spiked × 100%. The recoveries of the four analytes were between 96.3% and 100% (Table 3), which showed good analytical characteristics of this method.

### 3.5. Application of Method

To validate the method, three batches of* Ardisia japonica* extract samples were extracted using the optimized method.  Table 4 showed the content of all of the four analyses. For the four compounds, the average content of bergenin was the highest, between 3.07 mg g^−1^ and 11.77 mg g^−1^. The content of gallic acid ranged from 0.16 mg g^−1^ to 0.37 mg g^−1^, the content of quercitrin was between 0.21 mg g^−1^ and 0.88 mg g^−1^, and the content of embelin was changed from 0.66 mg g^−1^ to 2.08 mg g^−1^, respectively. The average of contents of the four analyses in the* Ardisia japonica* extract samples was different due to the difference of growth place of the raw materials. The average of the four analyses content was the highest of the herb grown in Hubei.

### 3.6. Comparison with Conventional Methanol Method

The chromatograms using different extraction methods for the same sample are compared. It was found that the chromatographic signals obtained by the CPE method were higher than those by the conventional methanol extraction method (Pharmacopeia of China 2015) because of its good enrichment capacity (Table 4). At the same time, peak areas of four components increased nearly 3 times by using cloud point preconcentration than those by using single micellar extraction (the enrichment ratio increased nearly three times) (Figure 6).

## 4. Conclusion

An environmentally friendly micelle-mediated extraction and cloud point preconcentration method was developed and validated for analysis of the four bioactive compounds in* Ardisia japonica* samples by using Genapol X-080 nonionic as extraction solvent. The optimal concentration of Genapol X-080, solution pH, liquid/solid ratio, ultrasonic time, concentration of NaH_2_PO_4_, temperature, and time of water bath were 4% (v/v), pH 5.0, 200 : 1 (mL/g), 40 min, 35% (w/v), 60°C, and 10 min, respectively. Under these conditions, high yield of the four compounds was obtained, which was higher than those with methanol method. It was proved that the method not only is simple, green, and reliable but also can obtain high enhancement factors and low limits of detection for the studied of four compounds of* Ardisia japonica*. The inexpensive Genapol X-080 solvent was used to extract and preconcentrate the trace bioactive compounds at low concentrations in the matrixes. It was concluded that micelle-mediated extraction and cloud point preconcentration will maintain a promising role in extraction and purification of active compounds from herbs medicines.

## Figures and Tables

**Table 1:** Calibration curve, linear range, LOD, and LOQ of the investigated analytes.

Analytes	Calibration curve	Linear range (*µ*g mL^−1^)	*r* ^2^	LOD (*µ*g mL^−1^)	LOQ (*µ*g mL^−1^)
Gallic acid	*y* = 19752*x* + 5015.7	0.4–50	0.999	0.013	0.04
Bergenin	*y* = 5179.4*x* + 29596	4–500	0.9988	0.012	0.04
Quercitrin	*y* = 12748*x* − 6495.3	1–50	0.9961	0.010	0.03
Embelin	*y* = 15290*x* + 4114.9	4–250	0.9964	0.016	0.04

**Table 2:** Precision and accuracy of the method (*n* = 6).

Analytes	Assay	Concentration (*µ*g mL^−1^)	Accuracy (%)	Precision (RSD%)
Gallic acid	Intra-assay	0.4	96.5	4.96
		2	97.9	0.79
		10	98.6	0.13
	Inter-assay	0.4	97.1	3.65
		2	97.7	0.93
		10	97.4	0.11

Bergenin	Intra-assay	4	97.7	0.47
		20	98.7	0.03
		100	98.7	0.03
	Inter-assay	4	97.6	0.44
		20	98.4	0.01
		100	97.0	0.02

Quercitrin	Intra-assay	0.4	98.6	4.10
		2	95.9	1.19
		10	100.0	0.25
		0.4	98.4	4.23
	Inter-assay	2	98.5	1.30
		10	97.8	0.17

Embelin	Intra-assay	1	98.2	2.53
		10	97.8	0.21
		50	97.9	0.03
		1	98.4	0.46
	Inter-assay	10	96.8	0.12
		50	97.3	0.03

**Table 3:** Recovery (*n* = 6) for four analytes in samples.

	Sample^1^ (*µ*g)	Spiked (*µ*g)	Determined^2^ (*µ*g)	Recovery (%)	RSD (%)
Gallic acid	0.82	0.80	1.59	96.3	4.22
Bergenin	21.07	21.00	41.66	98.0	3.21
Quercitrin	1.39	1.40	2.77	98.6	1.28
Embelin	2.22	2.24	4.46	100	3.51

^1^The amounts of analytes in plant samples extracted by ultrasonic assisted micellar extraction and then preconcentrated by cloud point extraction. ^2^The amounts of analytes in the spiked plant samples extracted by ultrasonic assisted micellar extraction and then preconcentrated by cloud point extraction.

**Table 4:** The concentrations (mg g^−1^) of four analytes in the three batches of *Ardisiajaponica* from different area (mg g^−1^) (*n* = 3).

Analytes	Hubei	Hunan	Guizhou
Gallic acid	0.29 ± 0.01	0.16 ± 0.00	0.37 ± 0.01
	0.18 ± 0.00	0.11 ± 0.01	0.21 ± 0.02
Bergenin	11.77 ± 0.40	3.07 ± 0.05	10.28 ± 0.28
	10.07 ± 0.11	2.44 ± 0.17	7.04 ± 0.50
Quercitrin	0.88 ± 0.01	0.21 ± 0.00	0.56 ± 0.01
	0.85 ± 0.02	0.16 ± 0.02	0.51 ± 0.04
Embelin	1.18 ± 0.01	0.66 ± 0.01	0.92 ± 0.00
	1.07 ± 0.03	0.66 ± 0.10	0.97 ± 0.02

*Note*. Upper line: micelle-mediated extraction and cloud point preconcentration method. Lower line: conventional methanol extraction method.
